# Inconsistent eating time is associated with obesity: A prospective study

**DOI:** 10.17179/excli2021-4324

**Published:** 2022-01-14

**Authors:** Darbaz Adnan, Jonathan Trinh, Faraz Bishehsari

**Affiliations:** 1 Rush Center for Integrated Microbiome and Chronobiology Research, Rush University Medical Center, Chicago, IL 60612, USA; 2 Department of Internal Medicine, Division of Gastroenterology, Rush University Medical Center, Chicago, IL 60612, USA; 3 Department of Anatomy and Cell Biology, Rush University Medical Center, Chicago, IL 60612, USA

**Keywords:** food timing, obesity, circadian, lifestyle, Body Mass Index

## Abstract

Obesity is characterized by an accumulation of redundant body fat linked to metabolic dysregulation and low-grade systemic inflammation. Lifestyle choices are imperative determining factors of obesity. The contemporary lifestyle is associated with behaviors that disrupt circadian rhythms, impacting metabolic homeostasis. Our animal and human studies suggest that circadian phenotypes could be related to the risk of metabolic dysregulation and obesity. The purpose of this study is to examine the role of inconsistent eating habits on body weight in adults. Individuals who presented for colon cancer screening were enrolled. Subjects received structured questionnaires to capture 7-day eating and sleeping times in a week prospectively. Bodyweight and height were extracted from medical records, and Body Mass Index (BMI) was calculated. Inconsistent eating times were defined as an average difference of >2 hours between the largest meal on weekdays and weekends. Forty-nine of the 61 (80.3 %) individuals enrolled in the study completed the questionnaires. The mean age and standard deviation (SD) were 60.8 (7.9), and 27 (55.1 %) were male. Subjects with inconsistent eating times had a significantly higher BMI (33.8 ± 3.6 SD, n = 9) than subjects who did not (27.5 ± 6.5 SD, n = 40; p = 0.001). The highest BMI was observed in subjects who ate inconsistently and late (35.8 ± 4.6 SD). In this cross-sectional study, time of eating habits was associated with BMI. Controlled cohort studies are needed to determine the potential link between eating time and the risk of obesity in the long term.

## Introduction

Obesity and overweight are chronic diseases defined by the accumulation of redundant body fat, which elicits chronic low-grade systemic inflammation and metabolic deterioration, contributing to alarming health problems (Must and McKeown, 2012[17]). The prevalence of obesity has nearly tripled globally over the past three decades, making the disease an epidemic (WHO, 2021[30]). This rapid rise in obesity, along with the changes in human lifestyle, suggests a significant role for environmental factors in obesity to such an extent that a modern human, if not consciously engaging in weight loss activities, would inevitably become obese (Seaman, 2013[21]). 

Lifestyle choices possess imperative roles in determining obesity. For example, many studies have demonstrated that excess intake of calorie-dense foods with a sedentary lifestyle is a primary contributing factor, but the etiology of obesity is particularly complex (Aronne et al., 2009[2]). For instance, obesity has been linked to other behaviors, such as disruption of biological rhythms. These rhythms are controlled by internal clocks in the human body orchestrated by the circadian system. The central clock is located in the brain and is regulated by the day/night cycles (Berson et al., 2002[3]). Disruption of this clock through alterations in light/dark cycles, such as in night-shift workers, has been associated with metabolic dysfunction (Swanson et al., 2016[24]; Takiishi et al., 2017[25]; Voigt et al., 2014[26], 2016[27]). 

The peripheral clock in the intestine, on the other hand, is essentially controlled by eating time (Stenvers et al., 2012[22]). We and others have shown that eating at times misaligned with the light/dark cycles, such as during and close to rest time, leads to central-peripheral dyssynchrony and circadian disruption in mice (Bishehsari et al., 2020[5]). Furthermore, our recent animal study illustrated that this discordance between eating time and light cycle with irregular eating habits between weekdays and weekends predisposes mice to weight gain. Such a behavior is a relevant characteristic of the modern human lifestyle since individuals commonly consume larger meals in the evenings closer to rest/dark phases (Takiishi et al., 2017[25]; Voigt et al., 2014[26], 2016[27]). However, the role of consistency in mealtimes has yet to be thoroughly explored. Here, we aimed to examine if inconsistent eating times are associated with obesity.

## Materials and Methods

We prospectively enrolled individuals who presented to the Gastroenterology Clinic at Rush University Medical Center (RUMC) for colon cancer screening. The study was carried out according to The Code of Ethics of the World Medical Association (Declaration of Helsinki) by obtaining informed consent, and the study protocol was approved by RUMC Institutional Review Board (IRB) (ORA # 14112503). Subjects with a history of Lynch syndrome, familial adenomatous polyposis, a recent history of cancer, or inflammatory bowel disease were excluded. Chronic comorbidities, identified through structured questionnaire or review of subject EMR that could affect food/sleep timing were excluded (i.e., chronic stage 3 or worse renal failure, cirrhosis, advanced neurological conditions [e.g., Parkinson's disease, MS, epilepsy], psychological disorders [e.g., PTSD, major depression], sleep apnea, restless leg syndrome, inpatient status, advanced cardiac failure [NY classification stage III/IV]).

After obtaining written informed consent from each subject, a 7-day questionnaire regarding eating and sleeping time was completed during their routine week (i.e., not traveling). Experts in gastroenterology and nutrition have designed the questionnaire at Rush University Medical Center (Chakradeo, 2018[9]). Subjects were also presented with questionnaires on demographics, medical history, and brief dietary intake characteristics for significant food categories that are linked to obesity and colorectal cancer (red meat, vegetables, fruit, whole grains, fried foods, and pickled foods).

The study subjects were educated on the questionnaire by a research coordinator during their research visit. They were contacted at home as well to address any of their potential questions on the questionnaire. The questionnaire was collected when the subject presented for his/her colonoscopy appointment. Sixty-one individuals were enrolled; only 49 individuals, 27 males (55.1 %) and 22 females (44.9 %) completed the study. The 7-day food structured timing questionnaire captured reporting time of self-defined eating events (breakfast, lunch, dinner, and snacks) on both work and free days (Chakradeo, 2018[9]).

Inconsistent eating times were defined as a difference > 2 hours between the average times of the largest meal on weekdays versus weekends. Inconsistent eating times were previously shown to be associated with worsening inflammation among inflammatory bowel disease patients (Chakradeo et al., 2018[10]). Late eating was defined as those who ate on average ≤ 2 hours before going to sleep (St-Onge et al., 2017[23]).

Bodyweight and height were extracted from medical records, and Body Mass Index (BMI) was calculated (kg/m^2^). BMI was further categorized to underweight (< 18.5 kg/m^2^), healthy weight (18.5-24.9 kg/m^2^), overweight (25.0-29.9 kg/m^2^), and obese (>30.0 kg/m^2^) (WHO, 2021[30]) (Table 1(Tab. 1)). Completed questionnaires were collected at the time of the colonoscopy.

Descriptive statistics, Student's t-test, and ANOVA were used to analyze the data and determine significant differences between measurements using SPSS version 26. Figures were created using Graph Pad Prism 5.

## Results

We recruited 61 subjects, of which forty-nine individuals, 27 male (55.1 %) and 22 females (44.9 %), prospectively completed and returned the 7-day food timing structured questionnaire. The mean age of participants was over 50 (60.9 ± 7.9 SD) years, as we enrolled subjects who presented for colon cancer screening, which usually doesn't start until age 50 and older (Table 1(Tab. 1)).

**Figure 1:**
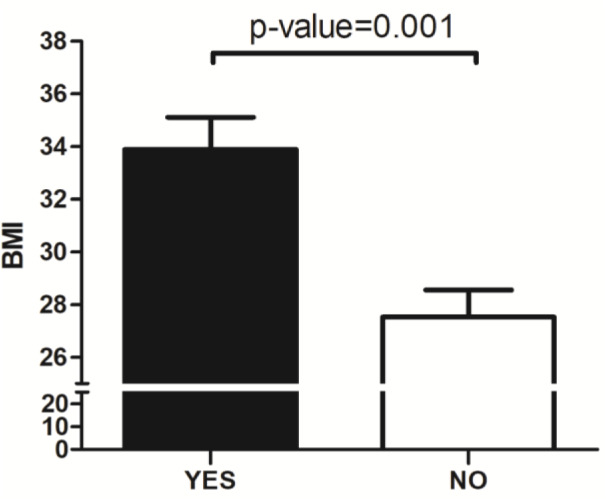
Inconsistent eating time and BMI mean with standard error of the Mean (SEM)

**Figure 2:**
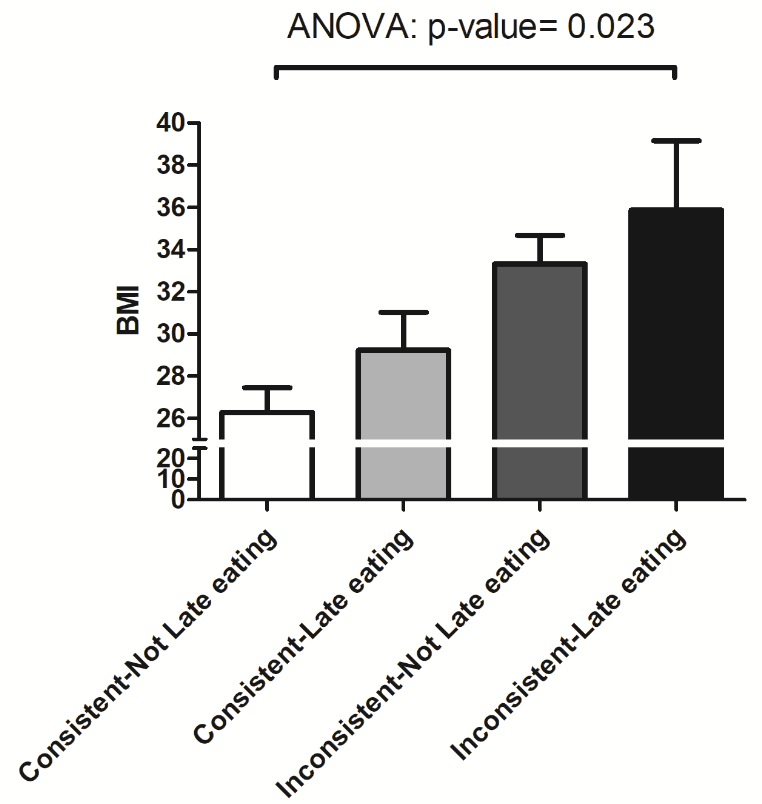
Subrgroup analysis of the time of eating habits. Mean + SEM

Subjects with inconsistent eating times had a significantly higher BMI (33.8 ± 3.6 SD, n = 9) than subjects who did not (27.5 ± 6.5 SD, n = 40; p = 0.001) (Figure 1(Fig. 1)). There was no significant difference in the diet type between these two groups (shown in Table 2(Tab. 2)). Individuals with late eating habits had an overall higher BMI (29.9 ± 7.4 SD) compared to those who did not eat late (27.9 ± 5.9 SD), but this did not reach statistical significance (p = 0.303). Comparison of the subgroups based on the time of eating habits (consistent/inconsistent * late/Not late eating) revealed a significant increase in the BMI of the following groups: subjects who ate consistently and early (26.2 ± 5.6 SD), subjects who ate consistently and late (29.2 ± 7.4 SD), subjects who ate inconsistently and early (33.3 ± 3.5 SD), and subjects who ate inconsistently and late (35.8 ± 4.6 SD) (P = 0.023) (Figure 2(Fig. 2)).

See also the Supplementary data.

## Discussion/Conclusion

In this study, we prospectively investigated the possible association between eating time habits and BMI. Our findings suggest that abnormal eating patterns defined by inconsistent eating patterns, especially if combined with late eating habits, may contribute to obesity.

Circadian rhythms are essential in coordinating biological processes to environmental routines such as sleep-active and eating cycles (Bishehsari et al., 2016[6]). Nearly every cell in the body possesses a molecular clock, which is wired to the central clock in the brain that is regulated by light-dark cycles. However, the clock in our digestive tract could be directly controlled by food signaling (Scheving, 2000[20]). As such, abnormal food timing with respect to the host activity phase could cause central-peripheral dyssynchrony, leading to circadian rhythm disruption (Bracci et al., 2014[8]; Mohawk et al., 2012[14]; Wehrens et al., 2017[29]). We have shown that wrong time feeding in mice, where the food availability is restricted to the rest phase, causes circadian rhythm disruption (Bishehsari et al., 2020[5]). Of note, most of our human participants in this study consumed their largest meal close to their rest phase at night. This data is consistent with studies that employed total energy intake distribution patterns and found dinner to be the largest meal in the majority of U.S. adults (Kahleova et al., 2017[12]). Although late eating alone was associated with higher BMI in our series, the difference did not reach statistical significance, likely due to our relatively small sample size, particularly in the category of early eaters.

We found that individuals with inconsistent eating patterns had higher BMI than those with consistent eating time, while the food components were comparable between groups. This is consistent with the results of our recent study wherein we observed that behavioral circadian phenotypes such as irregular food timing were associated with elevated BMI (Alsayid et al., 2021[1]). Interestingly, we observed a stronger association of inconsistent eating with elevated BMI in those who had a late eating habit (Figure 2(Fig. 2)). Although our study's association of time of eating habits with obesity is novel, it is consistent with the accumulating evidence that features of circadian disruption, such as light/dark shift or later meals, are associated with obesity (Bo et al., 2014[7]; Leproult et al., 2014[13]; Morikawa et al., 2007[16]). Our data is the first to suggest an association of obesity with the meal time's consistency, assessed over a week. All the participants recorded their food timing data prospectively for seven days, which minimized any recall bias inherent to retrospective studies. While data on inconsistent meal timing is scarce, inconsistent sleep timing, defined as > 2 hrs of difference in sleeping times between weekdays and weekends (i.e., social jet lag), is a common habit associated with circadian disruption and a higher risk of obesity and metabolic diseases (Roenneberg et al., 2012[18]). The observed association of the inconsistent meal timing with higher BMI by a prospective 7-day questionnaire is consistent with our recent observation using a food timing screener questionnaire (Alsayid et al., 2021[1]).

How exactly inconsistent eating patterns contribute to obesity requires further investigation. Our findings in animal models suggest that abnormal eating time could result in circadian dyssynchrony and gut dysbiosis, which can lead to intestinal barrier disruption and metabolic dysfunction (Bishehsari et al., 2021[4]). Other potential mechanisms can include food timing effect on hormonal regulation of energy hemostasis and metabolic regulation (Bishehsari et al., 2020[5]; Johnston et al., 2016[11]; Moossavi et al., 2019[15]; Scheer et al., 2009[19]; Wang et al., 2014[28]). 

In this proof-of-concept study, several limitations exist. For example, other unrecorded confounding factors (i.e., physical activity, detailed dietary intake, etc.) could account for the elevated BMI in the subjects who reported inconsistent eating patterns. Additionally, the relatively small sample size prevents causal or generalizable conclusions. As a result, this preliminary data calls for future controlled cohort studies using larger samples to examine the potential link between abnormal eating patterns and predisposition to obesity in the long term. 

## Declaration

### Acknowledgment

We thank Erika Davis for her assistance in the human studies.

### Conflict of interest statement

The authors declare that they have no conflict of interest.

### Funding sources

F.B. is supported by NIH: AA025387 grant.

### Author contributions

D.A. recruited, collected, and prepared the dataset on human subjects, analyzed the data, and helped with writing the manuscript. J.T. helped with data analysis and manuscript writing. F.B. designed the study, analyzed the data and drafted the manuscript. All authors revised and approved the final version of the manuscript.

### Data availability statement

Data is available and will be provided upon request.

## Supplementary Material

Supplementary data

## Figures and Tables

**Table 1:**
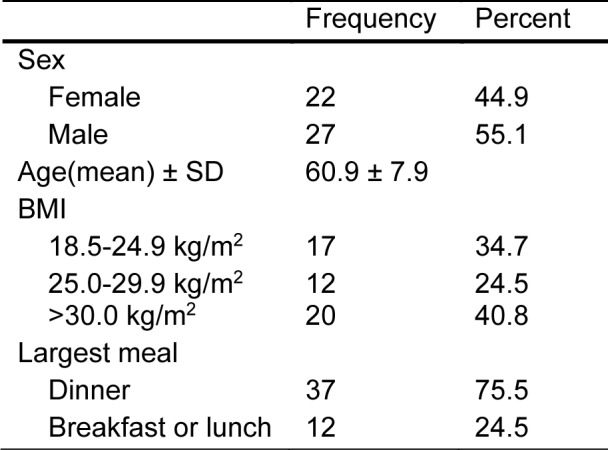
Characteristics of the study population

**Table 2:**
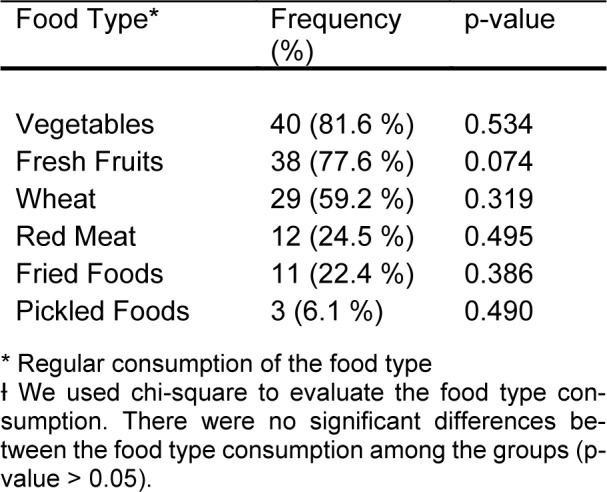
Food type consumption
